# Presumptive bacteriological diagnosis of spondylodiscitis in infants less than 4 years by detecting K. kingae DNA in their oropharynx: Data from a preliminar two centers study

**DOI:** 10.3389/fped.2022.1046254

**Published:** 2022-12-09

**Authors:** Moez Chargui, Andrzej Krzysztofiak, Paola Bernaschi, Giacomo De Marco, Benoit Coulin, Christina Steiger, Romain Dayer, Dimitri Ceroni

**Affiliations:** ^1^ Unité D'orthopédie Pédiatrique et de Traumatologie Infantile, Service de Chirurgie Pédiatrique, Hôpitaux Universitaires de Genève, Genève, Switzerland; ^2^ Infectious Diseases Unit, Academic Department of Pediatrics, Bambino Gesù Children's Hospital (IRCCS), Rome, Italy; ^3^ Microbiology Unit, Bambino Gesù Children's Hospital (IRCCS), Rome, Italy

**Keywords:** spondylodiscitis, kingella kingae, oropharyngeal, swab, presumptive, diagnosis

## Abstract

**Background and Objectives:**

Most cases of spondylodiscitis in children aged between 6 and 48 months old could be caused primarily by K. kingae. The present prospective study aimed to determine whether an innovative and indirect diagnosis approach - based on detection of K. kingae DNA in the oropharynx of children with suspected spondylodiscitis – provides sufficient evidence that this microorganism is responsible for the infection.

**Methods:**

We prospectively analysed infants admitted for spondylodiscitis, considering above all the results of PCR realized in oropharyngeal swabs and in blood samples.

**Results:**

Four of the 29 performed K. kingae-specific real-time PCR assay in blood were positive (13.8%), whereas 28 of the 32 K. kingae-specific real-time PCR assay realized on throat swabs were positive (87.5%).

**Conclusions:**

This study demonstrates that performing oropharyngeal swab PCR is able to detect K. kingae in almost 90% of the toddlers with confirmed spondylodiscitis. That provides strong arguments for the hypothesis that K. kingae should be considered as the main aetiological pathogen to suspect in children between 6 and 48 months old with spondylodiscitis. Finally, it seems to us reasonable that oropharyngeal swab may become an early decision-making tool for the indirect identification of K. kingae in spondylodiscitis.

## Introduction

Childhood spondylodiscitis is a term used to describe a continuum of primary pyogenic spinal infections (PPSI), from discitis to spondylodiscitis, as well as vertebral osteomyelitis with occasional associated soft-tissue abscesses (1–3). PPSI are uncommon entities, and their incidences are estimated to be 1–2 cases per year per 32,500 pediatric hospital consultations or 1 in 250,000 of the pediatric population (4, 5). The pathophysiology of PPSI is becoming clearer and less controversial; most authors now consider them to be infectious processes of the endplates and adjacent intervertebral discs (6–9), making the theory that they are self-limiting inflammatory conditions now completely obsolete (4, 10–14). Three main clinical forms of childhood PPSI have been described according to patients' ages (2, 6, 12, 15, 16). The neonate form affects infants under 6 months of age, and is the most severe manifestation of the disease; these children often present with *Staphylococcus aureus* septicemia and multiple infectious foci. Fortunately, this is the rarest form of the disease (2). The infantile form concerns children from 6 months (corresponding to the end of maternally derived immunity) to 48 months old, and this age group represents 60%–80% of cases of childhood spondylodiscitis (2, 15). Some studies of this age group have suggested that *Kingella kingae* might frequently be the microorganism responsible for spinal infection (2, 15, 16). Finally, in the third form, affecting children above 4 years old, patients are more prone to being febrile, appearing ill, and suffering from vertebral osteomyelitis due to *S. aureus.* Results from laboratory tests related to PPSI (white blood cell [WBC] count, C-reactive protein [CRP] level, and erythrocyte sedimentation rate [ESR]) do not often contribute to the diagnosis since they usually appear normal or slightly elevated (2, 15, 17–19). Blood cultures usually constitute the only available means of guiding antimicrobial therapy. Unfortunately, however, they show a high percentage of negative results, ranging from 88%–100% (2, 10, 15, 17–20). Indications for more invasive procedures, such as a biopsy or needle aspiration, are not currently established, especially in young children (12). Furthermore, the literature shows that the success rates for identifying causative organisms *via* needle aspiration or open biopsy range from 0%–63% (2, 13, 16, 20–22). These interventions, therefore, cannot be regarded as reliable, standard diagnostic procedures due to their poor performance and inherent surgical and anesthetic risks.

Since the 1980s, the reported number of cases of osteoarticular infections (OAI) attributed to *K. kingae* (23–29) has increased to such an extent that it has even been hypothesized that most cases of spondylodiscitis in children aged between 6 and 48 months old could today be caused primarily by *K. kingae* (2, 15, 30–32). In this context, the present prospective study aimed to determine whether an innovative, indirect-diagnosis approach—based on the detection of *K. kingae* DNA in the oropharynx of children with a suspected OAI (33)—could provide sufficient evidence that this microorganism is responsible for spondylodiscitis.

## Materials and methods

After approval by their respective institutional review boards, we prospectively analyzed children from 6 to 48 months old (infants) admitted to two tertiary institutions (Geneva University Hospitals, Geneva; Bambino Gesù Children's Hospital, Rome) for spondylodiscitis between January 2013 and December 2021. The present study only looked at cases meeting the inclusion criteria used by Fernandez et al. (18): patients were considered to have spondylodiscitis if they presented with clinical findings compatible with that diagnosis in addition to abnormal radiographic images. The latter included radiographic images showing narrowing intervertebral spaces or abnormal magnetic resonance (MR) images of the spine demonstrating involvement between the intervertebral space and nonadjacent vertebrae of normal appearance (18). Apart from radiographic appearance, clinical parameters such as fever at admission, a febrile condition prior to admission, and the time elapsed between symptom onset and diagnosis were recorded for each patient. Biological workups included peripheral WBC count, left-band shift percentage, platelet count, CRP level, and ESR. Bacteriological investigations included blood cultures for all patients. Polymerase chain reaction (PCR) assays were also performed on blood samples. No diagnostic biopsy or needle disc aspirations were performed in this case series. Oropharyngeal swab real-time PCR assays were also performed for each child since it has been previously demonstrated that this simple technique for detecting *K. kingae* RTX toxin genes in the oropharynx provides strong evidence that this microorganism is responsible for a patient's OAI (33). On that point, it has been suggested that a compatible clinical and biological picture, coupled with a positive *K. kingae-*specific NAAA on an oropharyngeal specimen provided strong evidence that this microorganism was responsible for the OAI for children less than 4 years years (in which more than 90% of OAIs are caused by *K. kingae*). However, it be kept in mind that a positive test is not an irrefutable proof of the etiology of the disease, since around 10% of young children carries the organism. Contrariwise, failure to detect *K. kingae* deoxyribonucleic acid sequences when using this sensitive molecular test virtually rules out the organism as the aetiology of the OAI. Thus, it is essential to repeat once again that this new diagnostic strategy may apply uniquely to children under 4 years old who present with clinical pictures that may suggest an IOA but with little translation on blood tests.

For the diagnosis of spondylodiscitis, we considered MR imaging to be the most sensitive technique in the disease's acute stage and comparable to computed tomography in its chronic stage. Diagnoses were primarily made using MR imaging characteristics. Spondylodiscitis characteristically involves two vertebral bodies and their intervening disk. It typically displays low signal intensity on T1-weighted images, with a loss of definition of the vertebral endplate and the adjacent vertebral bodies, and high signal intensity on T2-weighted images. In the disk space affected, fluid-like signal intensity is seen on both T1- and T2-weighted images. Following the intravenous administration of a gadolinium-based contrast material, disk enhancement patterns may be seen, such as a homogeneous enhancement of most of the disk, patchy non-confluent areas of enhancement, and thick or thin areas of peripheral enhancement. Infected bone marrow also enhances diffusely after a contrast material is administered; contrast-enhanced, fat-suppressed MR images are especially useful in demonstrating this marrow abnormality (34–36).

### Microbiological methods

In Geneva, the blood culture media used were BD BACTEC™ Peds Plus™ media. A real-time PCR assay targeting the *K. kingae* gene's RTX toxin was used for blood samples and oropharyngeal swabs. This assay is designed to detect two independent gene targets from the *K. kingae* RTX toxin locus, namely *rtxA* and *rtxB*. Since September 2009, oropharyngeal swab PCR has been performed for children from 6 months to 4 years old since it has been demonstrated to be a simple technique for detecting *K. kingae* RTX toxin genes in the oropharynx, providing strong evidence that this microorganism is responsible for OAI—or even stronger evidence that it is not (15).

In Rome, the BD BACTEC™ Peds Plus™ media has also been used since 2011.

Broad-range bacterial PCR amplification (Qiagen) was performed using universal primers complementary to the constant regions from part of the gene coding for 16S rRNA for blood samples and oropharyngeal swabs, followed by direct sequencing of the amplicons. The next-generation sequencing platform Illumina was used in this study in Roma for performing the Broad range PCR assays.

### Data analysis and statistical analysis

Data recorded included patient demographics, duration of symptoms, temperature at admission, any febrile condition before admission, types of infection according to MR imaging, laboratory studies and results, and bacteriological studies and results. Data were interpreted as means ± SDs unless otherwise stated.

## Results

The present study included 32 children with spondylodiscitis (16 girls, 16 boys); 23 patients in Geneva and 9 in Rome (Table 1). One case in Rome was excluded since no oropharyngeal sampling was performed. Mean age at admission was 21.2 months old ±7.7 months. The mean delay between establishing the diagnosis of spondylodiscitis and initiating treatment was 20.9 ± 16.6 days from symptom onset; in 5 cases (15.6%), the delay was over 1 month. Thirty children were afebrile at admission (93.8%); 6 children (18.8%) had a documented prodromal illness involving a temperature higher than 38 °C during the weeks before their hospital admission. No patients presented with neurological symptoms on clinical examination. Due to the existence of *K. kingae's* strains producer of penicillinase, all children were treated either with amoxicillin + clavulanate or with ceftriaxone.

**Table 1:** Epidemiological, clinical, biological and bacteriological characteristics of spondylodiscitis in infants & children aged 6 months to 4 years.

	Descriptive data
Age, mean (SD)	21.2 (7.7) months
Gender	16 males/16 females
Delay in establishing diagnosis	20.9 (16.6) days
Temperature admission, mean (SD)	36.9 (0.8)°
CRP, mean (SD)	18.4 (10) mg/L
WBC count, mean (SD)	10.545 (3.731) per mm^3^
Platelet count, mean (SD)	440.438 (181.100) per mm^3^
ESR, mean (SD)	37.1 (18.6) mm/h
Positive blood cultures	0/32
Positive rtPCR *K. kingae* on blood	4/29
Positive rtPCR *K. kingae* on oropharyngeal swab	28/32

WBC counts were normal (<12,000 per mm^3^) in 11 children (34.4%), with a mean of 10,545 ± 3731 per mm^3^ and no left-band shifts. CRP levels were normal (≤10 mg/l) in 10 children (31.3%) at admission and averaged 18.4 ± 10 mg/l for the entire study population. In parallel, the mean ESR was 37.1 ± 18.6 mm/h, and it was greater than 20 mm/h in 26 cases (81.3%). Mean platelet count was 440,438 ± 181,100 per microliter and rose above normal values in 18 cases (56.3%). None of the 32 patients had a positive blood culture. However, of the 29 *K. kingae*-specific real-time PCR assays performed on peripheral blood, 4 (13.8%) were positive. Of the 32 *K. kingae*-specific real-time PCR assays performed on throat swabs, 28 (87.5%) were positive. All the negative throat swab PCR assays (4) came from the same hospital; in one case, the negative test was probably because antibiotic therapy had started before the test was performed. Overall, the microorganism responsible for the spondylodiscitis was only able to be extracted from blood investigations (blood cultures or PCR assays on blood) in 4 cases (all *K. kingae*). In 24 additional cases, the *K. kingae*-specific real-time PCR assays performed on throat swabs were positive and thus highly suggestive of spondylodiscitis due to this pathogen.

Both radiographs and MR images were available for all patients. No occurrences of isolated discitis were encountered in this case series: all children presented with spondylodiscitis. These spinal infections affected the cervical spine in 4 cases, the thoracic segment in 2, the thoracolumbar junction in 3, the lumbar spine in 18, and, finally, the lumbosacral junction in the remaining 6 cases. The lumbar spine was the most frequently affected segment (54.5%).

## Discussion

To the best of our knowledge, the present work represents the first prospective, consecutive case series study on spondylodiscitis in infants from 6 to 48 months old. Significantly, it is the only study to have examined the predictive role of using oropharyngeal swabs to diagnose spondylodiscitis due to *K. kingae*. It also provides important data about this disease in terms of epidemiology, clinical and biological presentations, and micro bacteriological etiology.

To begin with, our study confirmed that for most patients, an appropriate diagnosis of spondylodiscitis was made relatively late in the course of the disease. The mean delay in establishing a diagnosis of spondylodiscitis was 3 weeks (range 2–62 days); these results were consistent with other studies reporting delays from 4 to 6 months (2, 4, 9, 37, 38). Our results also emphasized that the lumbar region was the most common part of the spine affected by spondylodiscitis, as suggested in many other studies (2, 7, 8, 13, 16), reporting that more than 50% of cases of spondylodiscitis were located in the lumbar spine.

All the children examined had sustained spondylodiscitis since none presented with pure discitis. As Garron et al. suggested, we believe that the pure discitis reported by some previous authors could not have occurred, due to reasons of vascular anatomy (2, 16, 39). In young children like our patients, the metaphysis of the vertebral body exhibits a rich vascular ring, which creates an anastomosis with the adjacent metaphyseal vascular ring through several branches adjacent to the disk's posterolateral region. The vascular supply to children's discs is made up of vessels that come through the cartilaginous vertebral plate and into the disc ring. Thus, any bacterial infections in children are probably primarily located in the metaphyseal region of the vertebral body, with the microorganism first having to cross the cartilaginous vertebral plate, running through the surface of the disc *via* the anastomotic branches, then infecting the adjacent vertebral metaphysis, and finally reaching the disc space between the two vertebral bodies involved (2, 16, 39).

Clinically, less than 10% of patients with spondylodiscitis had a body temperature greater than or equal to 38 °C at admission. The study also highlighted that there are few moderate laboratory findings in cases of spondylodiscitis. Indeed, our cohort of children from 6 to 48 months old only presented with mild-to-moderate clinical and biological inflammatory responses to infection, with the consequence that they presented with few, if any, of the usual criteria suggestive of an osteoarticular infection. More than 65% of our patients had normal or near-normal WBC and CRP levels. ESR (abnormal values in 81.3% of cases) and platelet count (abnormal values in 56.3% of cases) seemed to be the most sensitive markers to inflammation when a PPSI was present. Our results are thus in line with existing published data, which suggest that an increase in WBC count above 12,000 per mm^3^ is rare and present in only about 35% of cases (2, 18, 39). Similarly, our results confirmed that ESR increases slightly, and ranges from 35 mm/h to 90 mm/h (our study's mean value was 37.1 mm/h), as suggested in some other studies (2, 16, 40).

The present study also confirmed that attempts to identify the causative microorganism responsible for spondylodiscitis through blood cultures frequently failed. Blood cultures in children with spondylodiscitis are usually sterile (2, 10, 17, 20, 41), and in our case series, none of the blood cultures taken during initial workups was positive. Only the use of a real-time PCR specific for *K. kingae* allowed us to detect this pathogen's DNA in any of the blood samples (13.8% of cases).

Last but not least, the present study was the first to demonstrate that performing an oropharyngeal swab PCR could detect the *K. kingae* RTX toxin gene in almost 90% of the toddlers with confirmed spondylodiscitis. All the negative cases occurred in Rome, where only a broad-range 16S rRNA test was performed. This may explain the higher rate of negative oropharyngeal swabs since this assay only exhibits a sensitivity of 300 colony-forming units, which is ten times less sensitive than a real-time, toxin-specific PCR assay.

This is robust support for previous studies in which we suggested that *K. kingae* DNA could be found in the oropharynx of children with an OAI, and it indicates that a throat swab could provide strong evidence that this microorganism is responsible for an infection (15). Together this provides solid arguments for suggesting that *K. kingae* should probably be the primary etiological pathogen suspected in children from 6 to 48 months old with spondylodiscitis. The indirect diagnosis of *K. kingae*'s involvement in spinal infections, by searching for this specific pathogen's DNA in the oropharynx of infants, is of great interest in these particular situations, and performing discal needle aspirations or biopsies for the investigation of spondylodiscitis in this every age group should probably no longer constitute the gold standard.

The present study was limited by the small sample of children from two different centers. Its main limitation was that very few of these patients underwent diagnostic biopsies or needle disc aspirations, and thus a definitive identification of the causative organism was seldom possible. These findings deserve further investigation in prospective multicenter studies before it can be claimed that *K. kingae* is currently the main pathogen for OAI. In addition, a newly identified *Kingella* species called K. negevensis is recognized to be an occasional player in bone and joint infections in early childhood. Unfortunately, *K. negevensis* harbors a constitutional RTX locus similar to that of *K. kingae*, and as a result, positive assays targeting *rtxA* and *rtxB* only cannot be considered definitive evidence of *K. kingae* infection. Thus, one may suspect that any cases of this study due to *K. negevensis* can be misidentified as *K. kingae* infections by our clinical microbiology laboratories.

In conclusion, the present paper suggests that clinical and laboratory findings are often normal in spondylodiscitis involving children aged from 6 to 48 months old. Attempts to identify the causative microorganism responsible for spondylodiscitis using blood cultures often fail, especially if molecular methods are not employed. This study provided robust arguments for the hypothesis that *K. kingae* should henceforth be considered as the primary etiological pathogen suspected in children from 6 to 48 months old with spondylodiscitis. In the near future, the results of an oropharyngeal swab PCR assay could become an early decision-making tool for the indirect identification and treatment of *K. kingae* spondylodiscitis in young children. However, these preliminary results need to be validated by further prospective multicenter studies. Finally, it is important to keep in mind that *K. negevensis* may have caused clinical conditions that have been misclassified as *K. kingae* infections. Future studies using molecular diagnostic approaches capable of detecting *K. negevensis* are necessary to clarify this possibility.

## Data Availability

The raw data supporting the conclusions of this article will be made available by the authors, without undue reservation.
