# Erratum: Presumptive bacteriological diagnosis of spondylodiscitis in infants less than 4 years by detecting K. kingae DNA in their oropharynx: Data from a preliminar two centers study

**DOI:** 10.3389/fped.2023.1170911

**Published:** 2023-03-03

**Authors:** Frontiers Production Office

**Affiliations:** Frontiers Media SA, Lausanne, Switzerland

**Keywords:** spondylodiscitis, kingella kingae, oropharyngeal, swab, presumptive, diagnosis

An Erratum on Presumptive bacteriological diagnosis of spondylodiscitis in infants less than 4 years by detecting K. kingae DNA in their oropharynx: Data from a preliminar two centers study By Chargui M, Krzysztofiak A, Bernaschi P, De Marco G, Coulin B, Steiger C, Dayer R and Ceroni D. (2022) Front. Pediatr. 10:1046254. doi: 10.3389/fped.2022.1046254

An omission to the funding section of the original article was made in error. The following sentence has been added: “Open access funding was provided by the University Of Geneva”.

The original version of this article has been updated.

